# Research progress and application strategies of sugar transport mechanisms in rice

**DOI:** 10.3389/fpls.2024.1454615

**Published:** 2024-08-21

**Authors:** Jun Li, Changcai He, Shihang Liu, Yuting Guo, Yuxiu Zhang, Lanjing Zhang, Xu Zhou, Dongyu Xu, Xu Luo, Hongying Liu, Xiaorong Yang, Yang Wang, Jun Shi, Bin Yang, Jing Wang, Pingrong Wang, Xiaojian Deng, Changhui Sun

**Affiliations:** ^1^ State Key Laboratory of Crop Gene Exploration and Utilization in Southwest China, Rice Research Institute, Sichuan Agricultural University, Chengdu, China; ^2^ College of Agricultural Science, Panxi Crops Research and Utilization Key Laboratory of Sichuan Province, Xichang University, Liangshan, China; ^3^ Mianyang Academy of Agricultural Sciences, Crop Characteristic Resources Creation and Utilization Key Laboratory of Sichuan Province, Mianyang, China

**Keywords:** rice (*Oryza sativa* L.), carbohydrates, “source-sink” theory, sugar transporters, regulatory pathway, high-yield

## Abstract

In plants, carbohydrates are central products of photosynthesis. Rice is a staple that contributes to the daily calorie intake for over half of the world’s population. Hence, the primary objective of rice cultivation is to maximize carbohydrate production. The “source-sink” theory is proposed as a valuable principle for guiding crop breeding. However, the “flow” research lag, especially in sugar transport, has hindered high-yield rice breeding progress. This review concentrates on the genetic and molecular foundations of sugar transport and its regulation, enhancing the fundamental understanding of sugar transport processes in plants. We illustrate that the apoplastic pathway is predominant over the symplastic pathway during phloem loading in rice. Sugar transport proteins, such as SUTs and SWEETs, are essential carriers for sugar transportation in the apoplastic pathway. Additionally, we have summarized a regulatory pathway for sugar transport genes in rice, highlighting the roles of transcription factors (OsDOF11, OsNF-YB1, OsNF-YC12, OsbZIP72, Nhd1), OsRRM (RNA Recognition Motif containing protein), and GFD1 (Grain Filling Duration 1). Recognizing that the research shortfall in this area stems from a lack of advanced research methods, we discuss cutting-edge analytical techniques such as Mass Spectrometry Imaging and single-cell RNA sequencing, which could provide profound insights into the dynamics of sugar distribution and the associated regulatory mechanisms. In summary, this comprehensive review serves as a valuable guide, directing researchers toward a deep understanding and future study of the intricate mechanisms governing sugar transport.

## 1 Introduction

In plants, the primary photosynthetic products are carbohydrates, mainly monosaccharides (glucose and fructose), disaccharides (sucrose), and polysaccharides (starch), providing essential energy sources and matter for living organisms’ survival and growth. In cereal production activities, harvesting more edible carbohydrates is the ultimate goal (Alexandratos and Bruinsma, 2012; Julius et al., 2017). For high-yield crop breeding, the “source-sink” theory proposed by Mason M.L. and Maskell E.J. in 1928 has become an essential guiding theory (Mason and Maskell, 1928). The transportation and distribution of carbohydrates are the most critical components in this theory. The “source” refers to organs producing and exporting carbohydrates, such as leaves. In contrast, the “sink” represents organs that receive these sugars and store them as starch, such as seeds. The “flow” corresponds to the organs involved in sugar transportation between the “source” and “sink,” notably the phloem in the stem. According to this theory, high-yield breeding primarily focuses on optimizing the relationships of source-flow-sink, involving the enhancement of source organs to produce more photosynthetic products, increasing the storage capacity of sink organs, and improving the transport capacity of flow organs (Julius et al., 2017; Durand et al., 2018; Zhang and Turgeon, 2018).

Rice (*Oryza sativa* L.) plays a vital role as a primary provider of carbohydrates in the human diet and feeds more than half of the world’s population. Enhancing yield per unit area remains the foremost objective in rice breeding. Since the first Green Revolution and the introduction of hybrid rice technology, there has been no significant breakthrough in rice yield for years. In agricultural production, some rice varieties have large sinks and potent sources. However, their grains are not filled fully after maturity, with a low seed-setting rate, which seriously affects yield due to poor “flow” capacity (Mohammad et al., 1993; Mathan et al., 2021a; Li et al., 2022e). Furthermore, a recent study has shown that a more robust flow function may be a critical factor in the higher yield of cultivated rice than wild rice. While wild rice exhibits a more vital photosynthetic capacity and outputs more sucrose in the leaves, a weaker vascular system and reduced sucrose transporter activity limit the efficiency of transporting sucrose to the grains and yields (Mathan et al., 2021a).

Over the past decade, research on “source” and “sink” has been more in-depth, with a large number of genes related to photosynthetic efficiency and panicle traits being cloned and studied (Chen et al., 2022). However, our understanding of “flow” regulation has lagged despite the progress in the sugar transport of rice. Therefore, it is critical to explore essential genes related to sugar transport, elucidate their functions, and uncover the regulatory mechanisms (Ma et al., 2023). In this review, we reviewed the research progress and outlined a research prospect for sugar transport to facilitate further advancements in guiding high-yield rice breeding.

## 2 Core carbohydrates for study: sucrose and starch

Sucrose and starch, which are fundamental components of the plant’s sugar metabolism, are central indicators in flow study. By closely monitoring the levels and distribution of sucrose and starch, researchers could gain valuable insights into the dynamics of sugar transport and allocation in plants.

Sucrose, produced by photosynthesis, is the most prevalent disaccharide in plants. It is the primary transport form of sugar over long-distance in the phloem. Subsequently, a portion is allocated to support growth and development, with the remainder stored as starch in the sink (Patrick et al., 2013; Li et al., 2022c). Sucrose is a non-reducing sugar, rendering it more resistant to degradation, particularly during long-distance transport within plant vascular systems. Its low viscosity guarantees swift flow during transportation with higher speeds ranging from 0.5 to 3 m·h^−1^. Furthermore, sucrose is known for its remarkable solubility, with phloem concentrations typically falling within 200 to 1600 mmol·L^−1^, creating exceptionally high osmotic potential (van Bel, 1996; Kühn et al., 1999). These characteristics are crucial for enabling the efficient long-distance transport of carbon assimilates as sucrose within plants.

Starch, a polysaccharide composed of glucose molecules, is the primary storage form of sugar in plants. Its glucose molecule arrangement facilitates efficient hydrolysis, releasing energy when needed. This characteristic enables plants to store energy during periods of abundant photosynthesis and utilize it to support growth when necessary (Vandeputte and Delcour, 2004; Bao, 2019).

## 3 Process of sucrose transport

**Figure 1:**
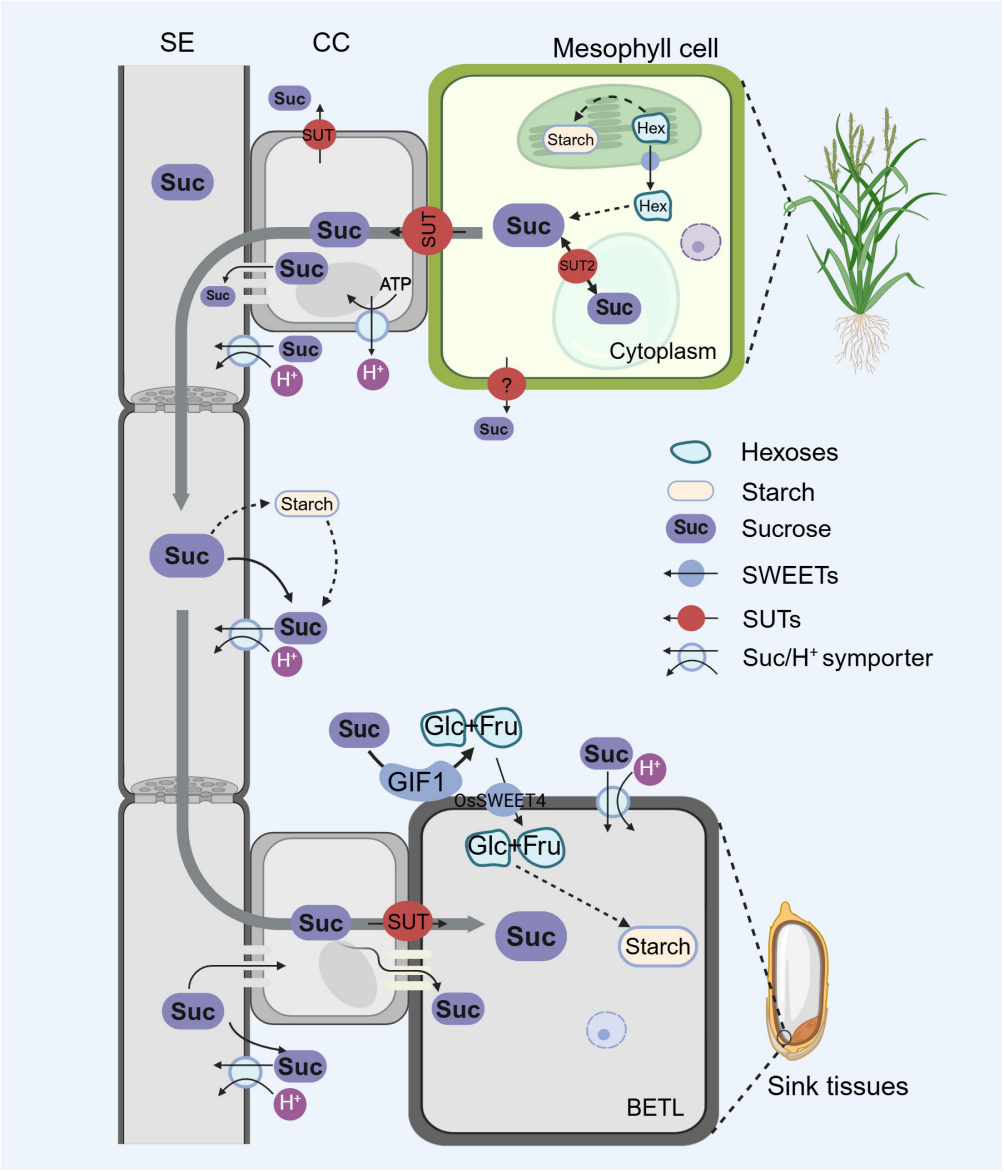
Long-distance transport of sucrose from source leaves to sink tissues, adapted from (Aoki et al., 2012). Sugars are produced in the mesophyll cells of source leaves, including hexoses, and are exported into the cytoplasm via sugar transport proteins. After undergoing a series of enzymatic catalysis reactions, hexoses are converted into sucrose. Sucrose enters the CC through symplastic and apoplastic pathways and is loaded into the SE of the phloem by these CC, then transported to sink tissues along the pressure potential gradient within the sieve elements. Sucrose is subsequently transported from the SE-CC complex to the surrounding parenchyma cells through plasmodesmata. In the apoplastic unloading pathway, sucrose is cleaved by cell wall invertase, generating hexoses such as Glc (Glucose) and Fru (Fructose). The produced Glc and Fru molecules are then transported into the BETL (Basal Endosperm Transfer Layer) for starch synthesis via the sugar transport protein OsSWEET4. Alternatively, sucrose could also be directly transported into storage cells. The figure was created with BioRender.com.

Sucrose transport in plants involves a highly coordinated process of three essential components: phloem loading, vascular transport, and unloading in the sink. These components ensure the successful delivery of sugars to where they are needed for growth, energy, and various metabolic processes (**Figure 1**). Furthermore, this extensive journey necessitates precise regulation and adaptability to meet plants’ growth and survival demands (Williams et al., 2000; Wen et al., 2022; Li et al., 2022c; Singh et al., 2023).

### 3.1 Phloem loading

The first crucial step in sucrose transport is loading it into the phloem, which occurs in source tissues such as leaves. Generally, plants have three major loading pathways: symplastic, apoplastic, and polymer trap pathways (Zhang and Turgeon, 2018).

#### 3.1.1 Symplastic pathway

The symplastic pathway could be the primary route initially taken for sucrose transport in the leaves. This pathway utilizes plasmodesmata, which are intercellular connections that provide channels for cytoplasmic continuity. By these channels, sucrose enters the phloem SE-CC (Sieve Element-Companion Cell) complex through passive diffusion along a concentration gradient. It does not require sucrose transport carrier proteins to cross membranes and does not consume energy, but it may face additional biological limitations, such as the distribution and connectivity of plasmodesmata (Miras et al., 2022). However, since sucrose could leak out of the cell during its transport in the phloem, sucrose transporters are still necessary to recover sucrose into the partition and maintain a higher chemical concentration gradient during symplastic transport (Braun et al., 2014).

#### 3.1.2 Apoplastic pathway

In some plants, the SE-CC complex lacks plasmodesmata connections with surrounding cells, so the apoplastic pathway is typically used for phloem loading. Unlike the symplastic pathway, which relies on plasmodesmata, the apoplastic pathway transports sugars through the cell apoplast. This pathway may offer greater flexibility for sugar transport, particularly during long-distance movement and in response to environmental changes (Aoki et al., 2012). In this pathway, the sucrose/H^+^ symporter is believed to mediate the transport of sucrose from the apoplast into SE-CC, and SWEET (Sugars Will Eventually be Exported Transporters) and SUT/SUC (Sucrose Transporters) are proved to be essential carriers. With the assistance of SWEET, sucrose is transported across membranes into the cell apoplast and then further transported to the SE-CC complex by SUT. This process entails active transport against a concentration gradient, which necessitates energy consumption, thus the efficiency of the apoplastic pathway may be influenced by factors such as pH (Hu et al., 2021).

#### 3.1.3 Polymer trap pathway

In the polymer trap pathway, sucrose is not directly loaded into the sieve tubes; instead, it is converted into oligosaccharides, such as raffinose and stachyose, in intermediary cells. These oligosaccharides then enter the SE-CC complex through larger plasmodesmata connections that rely on proton motive force for transport. Significantly, oligosaccharides like raffinose and stachyose could not diffuse back into mesophyll cells due to their larger molecular weight, thereby forming a kind of “trap” in the sieve tubes, which facilitates the loading of sugars (Comtet et al., 2017). This pathway plays a key role in ensuring that carbohydrates are efficiently conveyed to regions requiring them the most, thus facilitating growth and development.

### 3.2 Vascular transport

After phloem loading, sucrose enters the transport process within the vascular bundles. In general, vascular transport primarily involves long-distance translocation, during which metabolites are transported from the leaf vasculature to satisfy the diverse requirements of various plant tissues. The vascular bundles comprising the xylem and phloem tissues are the structural framework for metabolite transport. The phloem, primarily composed of sieve tubes and companion cells, forms an uninterrupted network throughout the plant, playing a crucial role in sucrose transport (White and Ding, 2023).

### 3.3 Unloading in sink

The final component is sucrose release from the phloem into sink tissues. Sink tissues can include developing grains, roots, and other organs that require a supply of sugars for growth and development. The phloem unloading process is also tightly regulated and can occur through symplastic, apoplastic pathways, or a combination of both. In the symplastic pathway, sucrose is transported from the SE-CC complex to the surrounding parenchyma cells through plasmodesmata. In the apoplastic unloading pathway, sucrose is subject to enzymatic cleavage by cell wall invertase, producing glucose and fructose. These resulting glucose and fructose molecules are then transported into the storage cells. Alternatively, sucrose can be directly transported into the storage cells (Braun, 2022; Sun et al., 2022; Wen et al., 2022; Li et al., 2022c).

## 4 Rice sucrose loading pathway

In grass crops, wheat, barley, and maize have been proven to use the apoplasmic loading pathway in sucrose transport, while it is still not entirely clear for rice (Julius et al., 2017). It has been reported that rice phloem sap collected by severing stylets of brown planthoppers contains primarily only one sugar, sucrose (Fukumorita and Chino, 1982). Therefore, rice does not employ the polymer trap pathway for sucrose phloem loading. The symplastic pathway or the apoplastic pathway could be used in rice.

### 4.1 Symplastic loading pathway in rice

Some studies suggest that rice has the fundamentals of cell structure for the symplastic loading of sucrose. Unlike maize, plasmodesmata could connect the parenchyma cells with companion cells in the leaf vascular bundles of rice (Botha et al., 2008; Eom et al., 2012). Using the characteristic that CFDA (Carboxyfluorescein Diacetate) can only be transported within the plastid after entering the plant body, the dye-feeding experiments showed that there is a continuous connection, known as symplastic continuity, between the phloem and the surrounding parenchyma cells (Scofield et al., 2007b). However, a later study reported that the frequency of such plasmodesmata connecting the cytoplasms of vascular bundles and companion cells accounted for only 1.4% of the total plasmodesmata measured across cell types (Braun et al., 2014). Though sucrose loading via a symplastic pathway may exist in rice, such loading capacity of sucrose may be relatively low. Therefore, a symplastic pathway may not be the primary pathway for sucrose transport in rice (Julius et al., 2017).

### 4.2 Apoplastic loading pathways in rice

Several recent studies support that sucrose phloem loading in rice predominantly takes the apoplastic pathway (Braun, 2022; Li et al., 2022b; Scofield et al., 2007a; Wang et al., 2021). Based on studies in Arabidopsis, it was hypothesized that if the membrane specificity of proton pyrophosphatases is localized within the SE-CC complex of leaves, there would be the potential existence of an apoplastic transport pathway in the plant (Gaxiola et al., 2012; Pizzio et al., 2015). In rice, immunogold labeling revealed that proton pyrophosphatases primarily localized to the plasma membrane of SE-CC complexes in the minor vein of rice leaves, reinforcing the notion of apoplastic loading. This interpretation was further supported by sucrose synthase-specific immunogold labeling at the SE-CC complex (Regmi et al., 2016).

Moreover, transgenic rice plants with overexpressed yeast invertase displayed reduced sucrose loading in the phloem, resulting in sucrose and starch accumulation in the leaves, akin to the growth-inhibited phenotype observed in *ossut1* mutants. The application of PCMBS (p-chloromercuribenzenesulfonic acid), an inhibitor of sugar transport protein function, notably hindered sucrose transport to rice phloem (Giaquinta, 1979; Wang et al., 2021). These results demonstrate the essential role of sucrose transport proteins in rice sucrose loading. Through electron microscopy, phloem tracer transportation, and ^13^C labeling, an additional study observed that the SE-CC complex did not exhibit a symplastic link with adjacent parenchyma cells in both leaves and stems. Moreover, the reduction in ^13^C isotope remobilization from leaves to stems and panicles upon PCMBS treatment indicated sucrose’s active transportation into the rice phloem (Li et al., 2022b). Therefore, rice is more inclined to use the apoplastic strategy for phloem loading.

## 5 Sucrose unloading pathway in rice

Till now, the sucrose unloading pathway in rice has been partially elucidated. Existing research indicates that, before unloading, the sucrose is initially enzymatically cleaved into glucose and fructose, with a crucial role played by a cell-wall invertase GIF1 (Grain Incomplete Filling 1) (Wang et al., 2008). Subsequently, glucose and fructose are transported into the endosperm via the sugar transporter protein OsSWEET4, which facilitates the transfer of glucose and fructose from the basal endosperm transfer layer to the endosperm for starch synthesis. Both *gif1* and *ossweet4* showed grain-filling defects, and *ossweet4* even resulted in defective seed filling, suggesting that the primary unloading pathway in rice grain might be the apoplastic pathway (Sosso et al., 2015).

## 6 Sugar transporters and their regulators in rice

Based on the above analysis, the primary pathway for sucrose transport in rice is the apoplastic pathway, underscoring the pivotal role of sugar transporters in this physiological process. In plants, sugar transport is facilitated by various sugar transporters, primarily including the SWEET family and MFS (Major Facilitator Superfamily). The MFS can be further categorized based on the substrate specificity into the DST (Disaccharide Transporter) family and the MST (Monosaccharide Transporter) family (Williams et al., 2000; Büttner, 2007; Deng et al., 2019; Zhou M. et al., 2023). In addition, there are still some other transporters specific to various forms of sugar, which are beyond the current scope of this review.

In rice, SWEETs and the disaccharide transporter SUTs, have been recognized as crucial facilitators in sugar transport (Eom et al., 2011; Julius et al., 2017; Mathan et al., 2021a). Although some other types of sugar-related transport proteins have been reported, such as OsMEX1 (Maltose Excess 1), OsTMTs (Tonoplast Monosaccharide Transporters), OsNope1 (No perception 1), OsBT1 (Brittle 1) and so on, further introduction is not provided here because these studies are currently neither in-depth nor systematic enough (Cho et al., 2010; Ryoo et al., 2013; Cakir et al., 2016; Nadal et al., 2017; Li et al., 2017; Song et al., 2020).

### 6.1 SUT family

SUT proteins have 12 transmembrane domains, forming a pore to transport sucrose across the plasma membrane (Riesmeier et al., 1992). The *SUT* family can be divided into three clades (Aoki et al., 2003). Type I *SUTs* are exclusive to eudicots, while type II and III *SUTs* are found in all plants. The rice genome encodes five *SUTs*; four are type II, and one (*OsSUT2*) is type III (Aoki et al., 2003). The five *SUTs* possess diverse roles in both sink and source tissues (Aoki et al., 2003). *OsSUT1*, *OsSUT3*, *OsSUT4*, and *OsSUT5* encode proteins in the plasma membrane, whereas *OsSUT2* encodes a protein in the vacuolar membrane (Aoki et al., 2003; Eom et al., 2011; Siao et al., 2011).

In Arabidopsis, AtSUC2 mediates efflux and retrieval via the apoplast pathway (Srivastava et al., 2008, 2009). Interestingly, *OsSUT1*, the type II SUT like *AtSUC2*, was able to complement the *atsuc2* mutant, indicating that *OsSUT1* is a functional homolog of *AtSUC2* for sucrose transporter (Matsukura et al., 2000; Furbank et al., 2001; Scofield et al., 2007a, 2007b; Hirose et al., 2010; Eom et al., 2016). *OsSUT1* is highly expressed in the endosperm, leaf sheaths, nodes, vascular parenchyma, and nucellar projection (Matsukura et al., 2000). However, it is curious that no visible phenotype was observed in *ossut1* knockdown plants at the beginning. Typical phenotypes of sugar transporter mutants, such as sugar accumulation within leaves and retarded growth, are not followed (Scofield et al., 2002; Hirose et al., 2010). Further study exhibited that it might be due to the low demand for sugar transport during the growth and development of rice in the greenhouse with low light. In the field-grown conditions, the *ossut1* mutant showed reduced growth and grain yield due to sucrose and starch accumulation in the leaves (Feng et al., 2018). Therefore, OsSUT1 could function to load sucrose from the apoplast into the phloem in rice leaves.

*OsSUT2* is highly expressed in lateral roots, inflorescence, stems, seed coats, leaf mesophyll, and bundle sheath cells but not in the veins (Aoki et al., 2003; Eom et al., 2011; Siao et al., 2011; Eom et al., 2012). *OsSUT2* may be involved in vacuolar-to-cytosolic sucrose translocation, influencing sucrose movement into the phloem (Sun et al., 2008; Eom et al., 2011; Siao et al., 2011; Eom et al., 2012). The mutant *ossut2* demonstrated distinctive growth-inhibited characteristics, shedding light on the crucial role of OsSUT2 in rice sugar transport mechanisms. Sugar export from the leaf was also reported to be significantly reduced in *ossut2* mutants, associated with notable accumulations of sucrose, glucose, and fructose in mature leaves (Eom et al., 2011, 2012). *OsSUT3* is preferentially expressed in pollen, indicating its involvement in pollen development and maturity rather than phloem loading in source leaves (Aoki et al., 2003; Scofield et al., 2007b; Li et al., 2020). *OsSUT4* shows expression dominance in leaves and stems. The *ossut4* mutant deficiency exhibited an accumulation of sucrose and starch in leaves and a significant reduction in net photosynthetic rate, which are the typical phenotypes of phloem loading obstruction in the vascular tissue (Mengzhu et al., 2020). *OsSUT5* exhibits the highest expression level in storage leaves (Aoki et al., 2003). Interestingly, through biochemical activity studies in *Xenopus* laevis oocytes, OsSUT5 was found to have a higher substrate affinity for sucrose and less substrate specificity than OsSUT1. Thus, OsSUT5 could potentially replace the function of OsSUT1 in sucrose phloem loading (Sun et al., 2010).

### 6.2 SWEET family

SWEETs are identified as sugar transporters containing seven transmembrane domains, which are present in all organism kingdoms (Yuan and Wang, 2013; Anjali et al., 2020; Singh et al., 2023). SWEETs mediate the bidirectional transmembrane transport of sugar substances, playing crucial roles in various physiological processes throughout plant growth and development. Among the 21 paralogs in rice, OsSWEET4 and OsSWEET11 are believed to play a predominant role in grain filling and yield formation (Zhou et al., 2014; Morii et al., 2020; Li et al., 2022d). Similar to OsSWEET4, OsSWEET11 is crucial in facilitating sucrose transport from maternal tissue to the maternal-filial interface in the stages of caryopsis development. Knockout of *OsSWEET11* led to decreased sucrose concentration in embryo sacs. Still, it increased the sucrose content and reduced starch levels in mature caryopses (Ma et al., 2017; Li et al., 2022d).

OsSWEET15 was found to share a similar function with OsSWEET11 in regulating the efflux of sucrose across the nucellar epidermis/aleurone interface. Notably, in the double mutant *ossweet11 15*, starch accumulation was observed in the pericarp, while a reduction in starch content was evident in the endosperm. This dynamic led to the development of shriveled caryopses (Ma et al., 2017; Yang et al., 2018). The single knockout mutants of *ossweet14* did not exhibit discernible phenotypic changes. In contrast, the double mutant *ossweet11 14* displayed considerably exacerbated phenotypes compared to the single-knockout mutants of *ossweet11*. These severe manifestations included a significant grain weight and yield reduction, a slower grain-filling rate, and an elevated accumulation of starch in the pericarp (Ma et al., 2017; Eom et al., 2019; Fei et al., 2021). Paradoxically, other studies have shown that *ossweet14* had a shorter plant phenotype and produced smaller seeds (Antony et al., 2010; Li et al., 2022d).

OsSWEET5, which acts as a galactose transporter, is believed to regulate plant growth and senescence by interacting with auxin signaling pathways (Zhou et al., 2014). Overexpressed *OsSWEET5* plants exhibited growth retardation and early senescence during the seedling stage. However, no discernible phenotype differences were observed in *OsSWEET5*-knockdown lines, suggesting the functional redundancy of galactose transporters existed in rice (Zhou et al., 2014). OsSWEET3a likely serves as a key player in facilitating the loading of glucose and GA (Gibberellin Acid) from the endosperm into the phloem for subsequent transport, thereby influencing early shoot development in rice. *OsSWEET3a* is primarily expressed in the vascular bundles of basal rice seedlings, while mutants lacking *OsSWEET3a* have significant deficiencies during germination and the initial stages of shoot development. Intriguingly, these impairments showed improvement when exogenous GA was introduced (Morii et al., 2020).

It is worth noting that overexpressing sugar transport proteins in a rough manner did not increase yield in rice. Some studies overexpressed *OsSWEET11* or *OsSWEET14*. However, the transgenic lines showed unfavorable phenotypes (Yuan et al., 2009; Gao et al., 2018; Singh et al., 2021; Kim et al., 2021b). The overexpression of *OsSWEET11* plants was markedly shorter and had fewer tillers than the wild type (Yuan et al., 2009), and *OsSWEET14* overexpressed plants resulted in a dwarf phenotype, reduced 1,000‐grain weight and grain number per panicle, and tillers (Kim et al., 2021b). Another study even co-expressed three sugar transporters, *OsSWEET11*, *OsSWEET14*, and *OsSUT1*, which got similar results to the plants that overexpress either *OsSWEET11* or *OsSWEET14* (Singh et al., 2021). These results showed that simply overexpressing sugar transporters does not improve the transport capacity of the flow but may have the opposite effect.

### 6.3 Transcriptional regulation of sugar transporters

Sugar transport through the phloem can be affected by many environmental factors that alter source-sink relationships, including abiotic and biotic, such as drought, saline-alkali, heat, mineral deficiency, pathogenic microbes, viruses, and so on. However, most of the exact mechanisms are far from being completely known at the molecular level (Krasensky and Jonak, 2012; Lemoine et al., 2013; Mathan et al., 2021a; Salvi et al., 2022).

**Figure 2:**
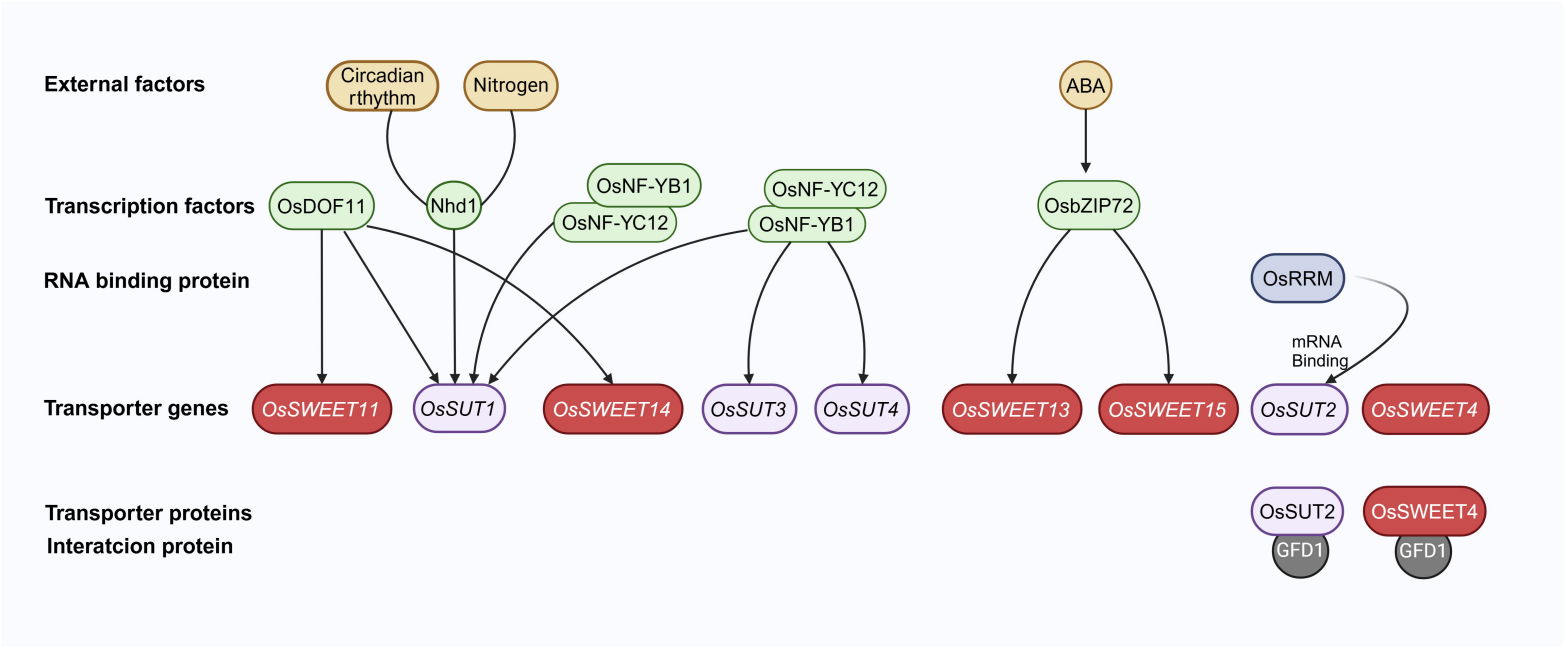
A simple regulation pathway of sugar transporters in rice. Transcription factors, such as OsDOF11, OsNF-YB1, OsNF-YC12, OsbZIP72, and Nhd1, directly regulate sugar transporter genes. These transcription factors are linked to the circadian clock, nitrogen absorption, and ABA stress signaling. The mRNA of *OsSUT2* could be directly bound by an RNA Recognition Motif containing protein, OsRRM. The GFD1 protein directly interacts with both OsSUT2 and OsSWEET4. Arrows represent positive regulation of sugar transporter genes. The figure was created with BioRender.com.

Recent reports indicate that sugar transporters are intricately connected with various regulatory pathways involving ABA (Abscisic Acid), GA, stress responses, nitrogen uptake, circadian rhythm, etc. Several transcription factors regulate the expression of rice sugar transporter genes, thereby controlling the sugar transport rate into various tissues (Yanagisawa and Sheen, 1998; Laloum et al., 2013; Li et al., 2013; Bai et al., 2016; Wu et al., 2018; Xiong et al., 2019; Liu et al., 2020; Sun et al., 2021; Zhang et al., 2021a; Kim et al., 2021b; Mathan et al., 2021b; Lee et al., 2022; Li et al., 2022a, 2023) (**Figure 2**).

DOF (DNA binding with one finger) transcription factors can recognize promoter elements containing (A/T)AAAG and subsequently regulate various aspects of plant development (Yanagisawa and Sheen, 1998). OsDOF11 is identified as a regulator of *SUT* and *SWEET* genes. It exhibits expression in vascular cells within photosynthetic organs and various sink tissues. The *osdof11* mutants displayed a semi-dwarf phenotype with reduced tiller numbers and smaller panicles, ultimately leading to a decreased sucrose transport rate compared to the wild type. Furthermore, the expression profiles of transporter genes, including *OsSUT1*, *OsSUT3*, *OsSUT4*, *OsSUT5*, *OsSWEET11*, and *OsSWEET14*, are significantly altered in the *osdof11* mutant. Chromatin immunoprecipitation assays have provided conclusive evidence for direct binding of OsDOF11 to the promoter regions of *OsSUT1*, *OsSWEET11*, and *OsSWEET14*. Interestingly, overexpression of *OsDOF11* enhanced resistance to sheath blight, while reducing yield production. Alternatively, tissue-specific activation of OsDOF11 via VP16 significantly improves yield production and enhances sheath blight, resistance, underscoring the pivotal role of tissue-specific in optimizing sugar transport-related genes (Li et al., 2013; Kim et al., 2021b).

NF-Y (Nuclear factor-Y) is a class of conserved transcription factors that binds to the promoter’s CCAAT box (Laloum et al., 2013). In rice, the *osnf*-*yb1* mutant exhibited chalky endosperm and defective grain-filling. RNA *in situ* hybridization assays displayed that *OsNF*-*YB1* was localized to the aleurone layer. EMSA (Electrophoretic Mobility Shift Assays) demonstrated that OsNF-YB1 could directly bind to the CCAAT box in the promoters of *OsSUT1*, *OsSUT3*, and *OsSUT4* (Bai et al., 2016). Another study showed that OsNF-YC12 was directly bound to the promoter of *OsSUT1* using various ChIP (Chromatin Immunoprecipitation) and Y1H (Yeast One-Hybrid assays). Furthermore, OsNF-YC12 could also physically interact with OsNF-YB1 (Xiong et al., 2019). Therefore, OsNF-YB1 and OsNF-YC12 dimers could regulate the expression of *OsSUTs* in the aleurone layer during the grain-filling stage.

*OsbZIP72* encodes a transcription factor responsive to ABA. Transactivation experiments have demonstrated that OsbZIP72 could directly bind to the promoters of *OsSWEET13* and *OsSWEET15* and promote their expressions. This finding suggests a plausible mechanism for regulating sucrose transport and allocation in response to abiotic stresses, and such a mechanism can potentially maintain sugar homeostasis in rice when faced with challenges such as drought and salinity (Mathan et al., 2021b).

An MYB transcription factor Nhd1 (N-mediated heading date-1), also known as a core clock component factor OsLHY (Late Elongated Hypocotyl), has been reported to regulate nitrogen uptake and flowering time (Sun et al., 2021; Zhang et al., 2021a; Lee et al., 2022; Li et al., 2022a). A subsequent study uncovered that the deletion of *Nhd1* led to the inhibition of OsSUT1 expression. Notably, when compared to the wild type, both *nhd1* and *ossut1* mutants displayed similar characteristics, including reduced plant height, decreased shoot biomass, and lower sucrose concentration. Intriguingly, the overexpression of *OsSUT1* could restore the impaired sucrose transport, partially alleviating the compromised growth observed in *nhd1* mutants. EMSA, transient transactivation assays, and ChIP-qPCR in rice leaf protoplasts provided evidence that Nhd1 could directly activate the transcriptional expression of *OsSUT1*, suggesting a conventional regulatory module involving *Nhd1* and *OsSUT1* that maintains carbon and nitrogen balance in rice (Li et al., 2023).

A recent study identified a novel mechanism for the expression regulation of rice sugar transporter. An OsRRM (RNA Recognition Motif) containing protein could interact with mRNAs of sugar transporter genes and positively regulate their expression levels, such as *OsSUT2* (Liu et al., 2020). To summarize, sugar transporter genes’ expression in rice is dynamically controlled by transcription factors and RNA-binding proteins to fine-tune sugar homeostasis (Dhungana and Braun, 2021).

### 6.4 Interacting proteins of sugar transporters

The transport activity of many transporters depends on the interactions of membrane proteins (Lalonde et al., 2010). For example, Arabidopsis nitrate transporter proteins NRT2.1 and NRT2.2 require interaction with the nitrate assimilation-related protein NAR2 to form a 150 kDa plasma membrane complex, which enables high-affinity nitrate transport (Quesada et al., 1994; Boone et al., 2007; Wirth et al., 2007; Lalonde et al., 2008; Jonikas et al., 2009; Yong et al., 2010; Jones et al., 2014; Sun et al., 2023). In Arabidopsis, the MIND (Membrane-based Interaction Network Database) revealed many interacting proteins of sugar transporter proteins (Cusick et al., 2005). However, their physiological and genetic functions are rarely reported in plants (Bitterlich et al., 2014; Jones et al., 2014; Abelenda et al., 2019; Xu et al., 2020; Wipf et al., 2021).

In rice, the interacting proteins of sugar transporters were unknown before a MATE (Multidrug and toxic compound extrusion) transporter protein GFD1 (Grain Filling Duration 1) was studied in our recent study (Sun et al., 2023). MATE transporter is one of the most prominent families of transporter proteins for cation transportation in most organisms (Omote et al., 2006; Takanashi et al., 2014; Upadhyay et al., 2019; Qin et al., 2021b; Zhang et al., 2021b; Zhou C. et al., 2023). Initially, due to the 12 transmembrane domains shared with SUT proteins, it was classified as a member of MFS (Marger and Saier, 1993; Omote et al., 2006). However, due to differences in protein sequence, it was later defined as an independent MATE transporter class (Brown et al., 1999).

In our recent study, GFD1 was identified as a MATE transporter protein that may be associated with sugar transport and starch accumulation in rice (Sun et al., 2023). The mutant *gfd1* disrupted the balance of carbohydrate distribution in the stem and grain but not in the leaves. The mutant showed a prolonged grain filling period, increased grain size, and reduced number of spikelets. Through Y2H (Yeast Two-Hybrid), LCI (Firefly luciferase Complementation Imaging assay), and BiFC (Bimolecular Fluorescence Complementation), it was proven that GFD1 could interact with two sugar transporters, OsSWEET4 and OsSUT2. Genetic analysis using double mutants indicated that GFD1 might control the grain filling period through OsSWEET4 and regulate grain size through OsSUT2, while working with OsSUT2 and OsSWEET4 to handle the number of grains per spike. However, the interaction effects on the functions of OsSUT2 and OsSWEET4 remain unclear (**Figure 2**).

On the other hand, in addition to the photosynthetic products from the leaves, the formation of rice grain yield also relies significantly on the starch stored in the stem before flowering. Stem starch can contribute up to 30% of rice yield, and this contribution rate increases with increased storage capacity, particularly under adverse environmental conditions. When rice photosynthesis is impaired, the stem becomes a primary source of assimilates for grain filling, significantly mitigating the negative effects of stress on yield (CocK and Yoshida, 1972). Furthermore, stored starch in the stem could also be a crucial factor contributing to the increased yield of cultivated rice compared to wild rice (Mathan et al., 2021a). Therefore, increasing the accumulation of starch in rice stems and improving the transport efficiency are essential to enhance rice yield. However, the molecular mechanism of starch accumulation in the stem remains unclear. Our study showed that GFD1 is an important regulatory factor for starch accumulation in the stem, and the deficient mutant *gfd1* significantly accumulates starch particles in the stem before grain filling (Sun et al., 2023). Therefore, GFD1 plays a significant role in the long-distance sugar transport within the stem.

## 7 Prospects for sugar transport study and application strategies in rice

**Figure 3:**
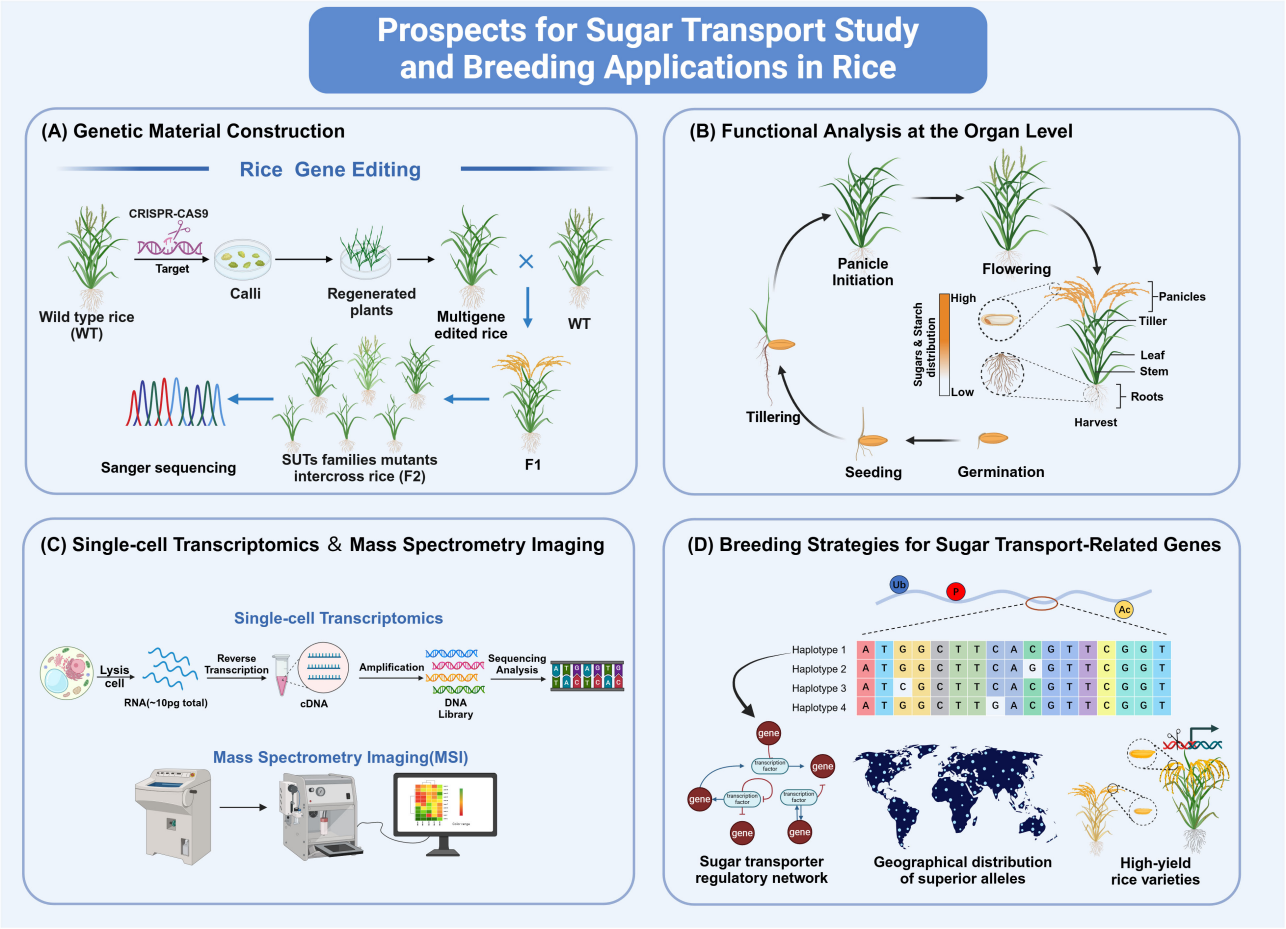
Prospects for sugar transport study and breeding applications in rice. **(A)** Gene editing technologies such as CRISPR/Cas9 have simplified the creation of mutants for SUT and SWEET families, facilitating the study of interactions between sugar transport proteins. **(B)** Measuring sugar and starch in tissues at various stages can reveal differences in their distribution. **(C)** Applying Single-cell transcriptomics and Spatial metabolomics allows for precise examination of multiple cells. **(D)** Using tissue-specific promoters, gene editing, and synthetic breeding with rice databases to select superior alleles can optimize sugar transport genes and boost rice yield, which is crucial for breeding. The figure was created with BioRender.com.

As biotechnology advances, focusing solely on studying sugar transport in organs such as roots, stems, leaves, and panicles is no longer sufficient. The development of single-cell sequencing and MSI (Mass Spectrometry Imaging) opens up new possibilities for a more precise understanding of the sugar transport mechanism. In order to facilitate a better understanding of the molecular mechanisms underlying sugar transport, we propose a study prospect here (**Figure 3**).

### 7.1 Genetic material construction

With the rapid advancement of gene editing technologies (Cong et al., 2013), obtaining a complete set of mutants for the genes of SUT and SWEET families has become more accessible. For instance, researchers have extensively employed CRISPR/Cas9 knockout techniques to generate mutants for most SWEETs and conducted comprehensive studies on these mutants (Li et al., 2022d). Furthermore, due to the redundancy of sugar transport proteins, it is now possible to create double, triple, or multiple mutants, thereby providing a rich genetic resource for investigating the precise interactions between these sugar transporters.

### 7.2 Functional analysis at the organ level

To elucidate the function of sugar transporters, measuring the sugar and starch content in different tissues at various developmental stages is essential. It could help uncover the differences in the distribution of sugars and starches across tissues (Sun et al., 2023). Such analyses will lay the foundation for further in-depth investigations into their microscopic functional differences using single-cell transcriptomics and spatial metabolomics techniques.

### 7.3 Single-cell transcriptomics

Our expanding knowledge highlights the remarkable heterogeneity in each cell, even within the same cell type. This intricate diversity and the various cell types in tissues amplify the need for spatially resolved single-cell omics techniques (Gilmore et al., 2019). In recent years, high-throughput single-cell transcriptomics has significantly progressed (Potter, 2018). Profiling multiple cells allows for a precise examination, unveiling unique insights into developmental processes, transcriptional pathways, and the molecular intricacies within tissues (Denyer et al., 2019). A notable investigation centered on vascular cells within Arabidopsis leaves, culminating in establishing a single-cell transcriptome of leaf vasculature. This comprehensive study successfully identified a minimum of 19 distinct cell clusters. Remarkably, among these clusters, PP cells exhibited a substantial enrichment of transporter genes, with prominent candidates including *SWEET11* and *SWEET12* (Kim et al., 2021a).

Leveraging this technology allows for the precise determination of the expression patterns of each sugar transporter gene at a single-cell resolution. Investigating these patterns is vital, encompassing the examination of single-cell expression patterns of sugar transporter genes, analysis of changes in downstream gene expression within sugar transporter mutants, and exploration of single-cell expression variations in upstream genes of the mutants. Furthermore, employing additional techniques such as *in situ* hybridization and immunofluorescence is pivotal and will substantially expedite the study of rice sugar transport (Qin et al., 2021b).

### 7.4 High-resolution spatial metabolomics technology

In recent decades, MSI has emerged as a powerful tool for non-targeted, label-free chemical imaging. It eliminates the need to pre-select specific molecular species for analysis, making detecting compounds without chemical modification or labeling agents possible. MSI encompasses various techniques such as MALDI-MSI (Matrix-assisted Laser Desorption Ionization Mass Spectrometry Imaging), DESI-MSI (Desorption Electrospray Ionization Mass Spectrometry Imaging), LAESI-MSI (Laser Ablation Electrospray Ionization Mass Spectrometry Imaging), SIMS (Imaging Secondary Ion Mass Spectrometry) and so on (Spengler, 2015; Buchberger et al., 2018).

By employing MSI, we can gain precise insights into the spatiotemporal distribution variations of sugars, including sucrose, fructose, and glucose (Hu et al., 2017). Utilizing MALDI-MSI, a recent study accurately depicted the spatial distribution of free soluble sugars (glucose, fructose, sucrose, and maltose) in rice seeds during the dough stage. These four free soluble sugars exhibited similar spatial distribution patterns, primarily concentrated in the seed cortex and embryo with high abundance (Zhao et al., 2023).

### 7.5 Breeding strategies for sugar transport-related genes

Merely elevating the expression of sugar transporters may not inevitably augment phloem transport capacity and has the potential to yield undesired outcomes. As demonstrated, the continuous overexpression of *OsDOF11* leads to decreased yield, while the tissue-specific activation of *OsDOF11* significantly enhances yield (Wu et al., 2018; Kim et al., 2021b). This underscores the crucial role of tissue-specific approaches when optimizing genes associated with sugar transport. Consequently, the strategic utilization of tissue-specific and site-specific promoters bears substantial significance.

In addition, the extensive publication of rice germplasm resources and genomic data has laid the foundation for identifying superior alleles of sugar transport-related genes (Wang et al., 2018; Qin et al., 2021a; Wang et al., 2023). Firstly, screening variant sites (including SNPs and InDels) within the promoters and coding regions of sugar transport-related genes and clustering analysis of their Haps (Haplotypes). Secondly, utilizing available databases of the yield-related traits, including 1000-grain weight, panicle grain number, and sugar distribution characteristics, such as 600 rice resources database (http://ricevarmap.ncpgr.cn/), analyzed their association with haplotypes of sugar transport-related genes for superior allelic variants identification. Thirdly, superior allelic variants enable functional validation and the creation of breeding materials in major rice cultivars. This could be achieved through precise gene editing and integrated breeding techniques, which facilitate the precise management of the balance of source-sink-flow.

In conclusion, the union of knowledge and methodological approaches concerning the cellular and tissue-level partitioning of sugar transporters, the elucidation of sugar transporters regulating networks, and their crosstalk with developmental mechanisms is imperative for a more sophisticated comprehension and precise manipulation of sugar transport mechanisms. This integrative perspective not only refines our grasp of the underlying regulatory models governing sugar transport but also prompts the optimization of rice germplasm. The strategic application of such knowledge is pivotal for enhancing rice yield potential and for stimulating its response to the environment.
